# Critical Assessment of Methods to Quantify Biofilm Growth and Evaluate Antibiofilm Activity of Host Defence Peptides

**DOI:** 10.3390/biom8020029

**Published:** 2018-05-21

**Authors:** Evan F. Haney, Michael J. Trimble, John T. Cheng, Quentin Vallé, Robert E. W. Hancock

**Affiliations:** Department of Microbiology and Immunology, Center for Microbial Diseases and Immunity Research, University of British Columbia, Vancouver, BC V6T 1Z4, Canada; evan@hancocklab.com (E.F.H.); mike@hancocklab.com (M.J.T.); jtj_cheng@yahoo.com (J.T.C.); valle.quentin@icloud.com (Q.V.)

**Keywords:** antimicrobial peptide, biofilm, high-throughput assay, antibiofilm peptides

## Abstract

Biofilms are multicellular communities of bacteria that can adhere to virtually any surface. Bacterial biofilms are clinically relevant, as they are responsible for up to two-thirds of hospital acquired infections and contribute to chronic infections. Troublingly, the bacteria within a biofilm are adaptively resistant to antibiotic treatment and it can take up to 1000 times more antibiotic to kill cells within a biofilm when compared to planktonic bacterial cells. Identifying and optimizing compounds that specifically target bacteria growing in biofilms is required to address this growing concern and the reported antibiofilm activity of natural and synthetic host defence peptides has garnered significant interest. However, a standardized assay to assess the activity of antibiofilm agents has not been established. In the present work, we describe two simple assays that can assess the inhibitory and eradication capacities of peptides towards biofilms that are formed by both Gram-positive and negative bacteria. These assays are suitable for high-throughput workflows in 96-well microplates and they use crystal violet staining to quantify adhered biofilm biomass as well as tetrazolium chloride dye to evaluate the metabolic activity of the biofilms. The effect of media composition on the readouts of these biofilm detection methods was assessed against two strains of *Pseudomonas aeruginosa* (PAO1 and PA14), as well as a methicillin resistant strain of *Staphylococcus aureus.* Our results demonstrate that media composition dramatically alters the staining patterns that were obtained with these dye-based methods, highlighting the importance of establishing appropriate biofilm growth conditions for each bacterial species to be evaluated. Confocal microscopy imaging of *P. aeruginosa* biofilms grown in flow cells revealed that this is likely due to altered biofilm architecture under specific growth conditions. The antibiofilm activity of several antibiotics and synthetic peptides were then evaluated under both inhibition and eradication conditions to illustrate the type of data that can be obtained using this experimental setup.

## 1. Introduction

Biofilms are clusters of single or multiple species of bacteria encased in a matrix composed of polysaccharides, proteins, and DNA that house and protect the bacteria from environmental pressures. Besides the physical barrier of the polymeric matrix, the bacteria within a biofilm undergo transcriptional changes to activate communication via quorum sensing and respond to the perceived stringent stressors and trigger resistance mechanisms that protect the cells from antibiotics and other antimicrobial threats [1]. Biofilms are ubiquitous in our environment [2,3,4], and it has been proposed that this is the natural growth state of bacteria [5]. Unfortunately, bacteria within biofilms are a significant concern as they are responsible for up to 65% of infections in humans [6,7] and are highly adaptively resistant (10–1000-fold) to conventional antibiotics [8]. Biofilm-associated infections pose a considerable problem to health care systems across the globe due to their persistence and the threat that they pose to the young, elderly, and immunocompromised. Unfortunately, no current antimicrobial therapies are available that specifically target bacteria within biofilms, which further limits the success of treatment and contributes to substantially increased healthcare costs and poor patient outcomes [9]. Therefore, the identification and the development of compounds that are capable of inhibiting biofilm growth or eradicating pre-formed biofilms is urgently required.

Host defence peptides (HDPs), which are also known as antimicrobial peptides, have garnered significant interest as potential alternatives to conventional antibiotics for the past two decades. More than two thousand peptides have been identified from various sources, including animals, plants, and bacteria [10], and many synthetic derivatives have been designed with enhanced antibacterial and anti-infective potency [11,12,13]. The seminal observation that the human cathelicidin LL-37 also possesses antibiofilm activity at sub-inhibitory concentrations [14] fuelled further interest in this class of molecules [15,16], and it is now commonplace for articles to include a measurement of antibiofilm activity as one of the parameters that is evaluated for a newly identified host defense peptide (HDP) sequence [17,18,19]. It is important to note however that the antimicrobial, immunomodulatory and antibiofilm activities of these peptides have distinct structure activity relationships, and thus here, we use the term antibiofilm peptides [20,21], based on the principle activity of interest. While the antibiofilm effects of peptides are of great interest with respect to their clinical development, there is at present no standardized assay to evaluate the antibiofilm activity of novel peptides. As a result, it is difficult to compare the literature-reported antibiofilm activities of such peptides, since each group uses different detection methods to quantify biofilm growth.

To rapidly develop and to screen peptides for their antibiofilm activity a quick, easy, reproducible, and inexpensive method is required. Unfortunately, when compared to minimum inhibitory concentration (MIC) determination, where the assay end point is readily determined due to the binary nature of growth versus no growth [22], the variability in biofilm architecture and the complexity of their development makes the interpretation of assays that are used to detect and monitor biofilm growth more challenging. Assays that stain the residual adhered biofilm biomass after incubation with a compound of interest are colloquially termed as BIC (biofilm inhibitory concentration), MBIC (minimum biofilm inhibitory concentration), MBEC (minimum biofilm eradication concentration), and MBC (minimum bactericidal concentration) assays [23,24,25,26]. These assays attempt to impose the concept of MIC onto biofilm inhibition and eradication without sufficient consideration of the major differences in the experimental setup and conditions. For example, in the standard MBIC assay, an antimicrobial compound of interest is added to a suspension of planktonic cells and then following incubation, the planktonic cells are discarded and the biomass that remains adhered to the assay plate is stained and quantified. Unfortunately, this assay cannot usually distinguish between planktonic killing by the antibiotic and specific antibiofilm effects, since bacteria are exposed to the compound of interest before they have a chance to adhere. Similarly, assaying residual bound bacteria using crystal violet (CV) has issues, since CV stains biomass rather than living bacteria, and thus dead bound bacteria will still be stained. Thus, it is important to assess the best method to monitor and to analyze biofilm growth in the presence of antibiofilm/antimicrobial agents.

There are many protocols that have been proposed to analyze relative biofilm formation [27]. Some seek to grow biofilms under conditions that allow for the constant flow of fresh media over the growing biofilm since biofilms that are grown in flow conditions are considered to be more physiologically relevant to natural biofilms and they provide valuable insights into biofilm morphology and structure. For example, BioSurface Technologies’ CDC Biofilm Reactor (Bozeman, MT, USA) grows biofilms with replenished medium on surface “coupons” under shear flow conditions [28]. Similarly, the Modified Robbins Device is a laminar flow chamber that houses suspended substrates for analysis of biofilm growth under experimental conditions [29,30]. Perhaps the most widely used method to grow biofilms under flow conditions are flow cell chambers assessed by visualization of the adhered biofilms using confocal microscopy [31]. While each of these methods are valuable tools for studying biofilms under controlled conditions, they all require specialized equipment, are technically challenging, and are not amenable to rapid high throughput assays to screen antibiofilm peptides or other antimicrobial compounds. Moreover, this concept of physiological relevance of biofilms grown in these flow settings is not a universal perspective and an alternative consideration is that within the human body, tissue embedded biofilms are not necessarily subjected to flow conditions.

Static biofilm assays typically allow for 96-well plate formats and are more amenable to high-throughput screening approaches. The Calgary biofilm device is an innovative approach that allows for biofilms to grow on pegs suspended from a specialized lid that fits on a standard 96-well microtitre dish [32]. This allows for the user access to remove pin-adherent biofilms from the well for increased accuracy in assessing colony forming unit (CFU) counts and CVstaining. The BioFilm Ring Test (BioFilm Control, Saint Beauzire, FR) involves growing bacteria in the presence of magnetic beads within the wells of a microtitre dish. Following growth and biofilm formation, the dish is placed onto a magnetic plate, which attracts the beads to the center of the well [33]. If a biofilm has formed, then the biomass prevents the beads from accumulating at the center of the well when being exposed to a magnetic field, and this can be quantified using a specialized plate reader. This assay is rapid and it requires no wash steps or dyes for analysis, but it can only measure biofilms that form sufficiently thickly on the bottom of the well of microtitre dishes. Similarly, the xCELLigence machine (ACEA Biosciences Inc., San Diego, CA, USA) is a versatile instrument that has been used to monitor biofilm formation in real time by measuring electric impedance due to biofilms that form on the bottom of specialized microplates [34]. Unfortunately, this experimental setup does not allow for measurements of biofilms that form on the side of the microplate well and the machine itself is costly as are its specialized electrode plates. All of these techniques fit the requirement for high-throughput workflows to assess biofilm growth, but the last two, in particular, require specialized detection equipment that may not be available to or affordable by each researcher.

Owing to their ease of use and relative low cost, dye-based methods are often used to quantify biofilm growth in static microplate assays. Dye-based methods such as safranin [35] as well as metabolic dyes, such as tetrazolium-based dyes [36,37] or rezazurin dye [38,39], and other fluorescent labels can be translated to high-throughput workflows in microtitre plates. However, the CV staining method, originally described by O’Toole and Kolter in 1998 to identify biofilm-deficient mutants [40], has become the “gold standard” for quantifying biofilms in a microtitre dish. It is an inexpensive assay that can be routinely performed with relative ease [41], can be used for both Gram-positive and Gram-negative organisms, and is suitable for qualitative and quantitative measurements of biofilms adhered to a variety of surfaces. The popularity of this method cannot be overstated, as the original paper has been cited more than 2100 times since it was published in 1998. CV is a dye that is familiar to all microbiologists since it is used in the Gram staining procedure, to stain Gram-positive bacteria purple since their thick peptidoglycan wall retains the bound CV dye, while the Gram-negative outer membrane excludes the dye [42]. In spite of its popularity, CV has certain drawbacks, including non-specific binding to anionic proteins and other negatively charged molecules, like capsules, lipolysaccharides, and DNA/nucleic acids, leading to an inability to distinguish between live and dead bacterial populations. These issues contribute to a large variability between samples that may complicate the interpretation of biofilm screening results.

Here, we have compared the various methods for quantifying biofilm growth, including CV staining, metabolic dyes, and CFUcounts, and discuss the strengths and the limitations of each. We examined the effect of growth media on the outcomes of biofilm growth using these various detection methods, revealing a large influence of medium composition on biofilm quantification that may often be overlooked in biofilm screening studies. In addition, we characterized biofilms that were grown on either glass or plastic surfaces, as well as under conditions of flow to demonstrate that this heterogeneity of biofilm growth is independent of the experimental setup. Then, we evaluated a standard assay [43] for assessing the inhibition of biofilm development by adding, to the wells of a microtitre plate, compounds of interest concomitantly with bacteria, and following overnight incubation characterizing the residual biomass using CV. This type of assay is common and it is the primary experimental setup that we have used in the past to screen synthetic peptides for antibiofilm activity [16,20,21].

Such biofilm inhibition assays are indispensable tools for identifying compounds that prevent initial attachment or interfere with the early stages of biofilm growth. However, in the context of a clinical infection, assays that capture the antibiofilm activity of compounds against pre-formed biofilms are also required, since these will presumably identify compounds with the potential to act on established biofilms. Few studies evaluate the effects of compounds on preformed biofilms, likely due to inherent technical difficulties in growing biofilms. Here, we describe an assay that is suitable for high-throughput workflows, which is capable of evaluating the capacity of antibiofilm peptides or other antimicrobial compounds to inhibit or to eradicate pre-formed biofilms. The proposed method combines the use of CV staining to measure biofilm biomass and a tetrazolium based metabolic dye to assess the residual viable metabolizing bacteria. Such an approach permits us to fully capture the types of activities that can be exerted by antibiofilm compounds, including biofilm dispersal and inhibition of biofilm growth. The proposed methodology can be used for both Gram-negative and Gram-positive organisms, is relatively simple to implement, uses a single 96-microtitre plate for biofilm growth and quantification, and it uses inexpensive and commonly used dyes.

Examples of inhibition and eradication assays are presented for both *Pseudomonas aeruginosa* and methicillin resistant *Staphylococcus aureus* (MRSA) biofilms evaluating the antibiofilm activity of various antibiotics and antibiofilm peptides and special considerations for each assay type are discussed. Importantly, the proposed antibiofilm screening methods provide reproducible workflows that are capable of assessing the inhibitory and eradication capacity of novel compounds that will be essential to identify antimicrobial agents that specifically target biofilms.

## 2. Methods and Materials

### 2.1. Source of Synthetic Peptides and Chemical Reagents

Synthetic peptides 1018 (VRLIVAVRIWRR-NH_2_) and DJK-5 (all d-amino acids, *VQWRAIRVRVIR*-NH_2_) were obtained from CPC Scientific (Sunnyvale, CA, USA) as 95% pure peptides as acetate salts. Peptide 3002 (ILVRWIRWRIQW-NH2) was obtained from Genscript (Piscataway, NJ, USA) also at 95% purity. Antibiotics and other chemical reagents were obtained from Sigma-Aldrich (St. Louis, MO, USA) or Thermo Fisher Scientific (Waltham, MA, USA).

### 2.2. Bacterial Strains and Growth Conditions

Bacterial strains used in this study included *Pseudomonas aeruginosa* strains PAO1 and PA14, and a clinical strain of methicillin resistant *Staphylococcus aureus* (MRSA, strain SAP0017, clinical isolate obtained from Tony Chow at Vancouver General Hospital). Several media were evaluated in this study, with many of them being modified forms of basal medium 2 (BM2) consisting of 62 mM potassium phosphate buffer, pH 7.0, 7 mM (NH_4_)_2_SO_4_, 0.5 mM MgSO_4_, and 0.4% glucose. Additional BM2 media were prepared altering the concentration of MgSO_4_ (2, 0.05, or 0.005 mM) or changing the carbon source (0.4% fructose, 0.4% sucrose, 0.4% galactose, 0.4% glycerol, 0.4% mannitol, 20 mM pyruvate, or 20 mM succinate). The effect of adding exogenous FeSO_4_ (1, 10, and 100 μM) as well as 10 μM CaCl_2_ to the original BM2 recipe, was evaluated (e.g., CaCl_2_). Other media included Luria Broth (LB, Thermo Fisher Scientific Cat# BP1427-500) and tryptic soy broth (TSB, BD Bacto, Thermo Fisher Scientific Cat# DF0370-17-3), made according to the manufacturers specifications and then evaluated by diluting in sterile water at various strengths (*v*/*v*: 100%, 50%, 10%, 2%, or 1%) or supplementing with different concentrations of glucose (1% or 0.1%). M9 minimal media (26 mM Na_2_HPO_4_, 22 mM KH_2_PO_4_, 9 mM NaCl, 19 mM NH_4_Cl, 0.4% glucose, 2 mM MgSO_4_, and 0.1 mM CaCl_2_) was also evaluated using a recipe that was adapted from Cold Spring Harbor protocols [44].

### 2.3. Biofilm Growth in Glass Tubes

To assess the effect of media composition on biofilm growth, *P. aeruginosa* PA14 and PAO1 were separately grown in glass tubes in several different media. Cultures were inoculated by adding 10 µL of an overnight culture of bacteria into 1 mL of sterile media, and the tubes were incubated statically at either 37 °C or at room temperature (~21 °C) for two days. Planktonic growth was documented photographically. The supernatant was then discarded and the adhered cells were rinsed three times with distilled water, and the tubes were patted dry on a paper towel. One mL of a 0.1% CV solution was added to each tube to stain the adhered biomass and the tubes incubated for 30 min at room temperature. The CV dye was discarded and the tubes were again rinsed three times with distilled water and were patted dry. Tubes were photographed to document the amount of biofilm that was adhered to the glass surface. One mL of 70% ethanol was then added to each tube to release the bound CV dye from the biofilm, and 100 µL of this was transferred to a 96-well plate for quantification at an absorbance of 595 nm (A_595_) on a plate reader. All of the growth conditions were evaluated in triplicate.

### 2.4. Biofilm Growth in Flow Cells

Biofilms were grown in flow chambers, as described previously [15,16], with slight modifications. Briefly, three-channel flow cell chambers (IBI Scientific, Peosta, IA, USA), were sterilized with a 10% bleach solution and rinsed with sterile water, followed by modified BM2 minimal medium for one hour. The chambers were inoculated with 400 μL of an overnight culture of bacteria grown in LB broth diluted to an optical density at 600 nm (OD_600_) of 0.005 in the appropriate media. The flow cells were then inverted for 3 h without flow to allow for bacterial adherence to the glass coverslip of the flow cell. Following bacterial adhesion, the appropriate BM2 medium was passed through the system at a constant flow rate of 2.4 mL/h for three days to allow for biofilm growth and maturation. Subsequently, the flow cells were flushed with medium for 5–10 s at the maximum flow rate for the peristaltic pump (~8.5 mL/min) in order to remove planktonic and un-adhered cells. The attached biofilms were stained with 1 μM SYTO-9 (from LIVE/DEAD BacLight Bacterial Viability kit; Molecular Probes, Eugene, OR, USA), diluted in 0.9% NaCl. Confocal microscopy was performed using a Zeiss LSM 800 microscope fitted with a 20x/0.8 Plan-APOCHROMAT objective and images captured using the Zen system software (Carl Zeiss Canada Ltd., Toronto, ON, Canada). The experiment was carried out at least twice and multiple images were captured per flow chamber. Image processing was performed with Zen software and a three-dimensional reconstruction of the Z-stack images was performed with the free ImageJ (version 1.51w, National Institutes of Health, Bethesda, Maryland, USA) software package Fiji [45,46].

### 2.5. Microtitre Biofilm Inhibition Assay

The inhibition of biofilm formation was assessed using methods that were described previously [20]. Briefly, 90 μL of a bacterial suspension (final OD_600_ = 0.01), prepared by diluting an overnight culture grown in LB broth into the medium of interest, was added to the interior wells of a 96-well polystyrene microtitre plate containing 10 μL of peptide at 10× the final desired concentration, or 10 μL of vehicle control. A diagram depicting a typical microtitre plate layout for this experiment is shown in Figure S1. The plates were incubated overnight at 37 °C under static conditions to allow for bacterial growth and biofilm maturation. The following day, bacterial growth was quantified by recording OD_600_ of each well using an Epoch Microplate Spectrophotometer (BioTek Instruments Inc., Winooski, VT, USA). The planktonic cells and the spent medium were discarded, and the adhered biomass was rinsed three times with distilled water. The biomass was stained with 0.1% CV solution for 20 min and then rinsed three times with distilled water to remove unbound dye. The bound CV dye was resuspended in 70% ethanol with gentle mixing and the A_595_ was recorded in the same sample plate. The amount of biofilm inhibition was calculated relative to the amount of biofilm that was grown in the absence of peptide (defined as 100% biofilm) and the media sterility control (defined as 0% biofilm). Results from at least three separate biological replicates were averaged.

### 2.6. Microtitre Biofilm Eradication Assay

To evaluate the effects of antibiotics and peptides on pre-formed biofilms, a method was devised employing a combination of CV staining as well as a metabolism-based tetrazolium dye. To establish biofilms, 100 μL of a bacterial suspension (OD = 0.01) in the media of interest was added to the interior wells of a 96-well microtitre plate. For each condition tested, two individual wells were prepared on separate microtitre plates in parallel, one to eventually be stained with CV and the other to be treated with a metabolic dye, triphenyl tetrazolium chloride (TTC). As in the biofilm inhibition assay, the wells on the edge of the plate were not used to grow biofilm samples (Figure S1). The plates were incubated overnight at 37 °C to allow for biofilm attachment and growth. The following day, the planktonic and unbound cells were aspirated from each well, and the adhered biofilm was rinsed three times with 150 μL of fresh sterile medium using a multi-channel pipette. Excess rinse medium was removed from each well by aspiration and then 180 μL of sterile media was added to each well, followed by 20 μL of a 10× concentrated solution of the peptide or antibiotic of interest, or 20 μL of vehicle controls. For samples that were being evaluated for their metabolic activity, sterile TTC was added to a final concentration of 0.05%. The plates were again incubated overnight at 37 °C under static condition. The following day, the cumulative bacterial growth consisting of both adhered biofilm and planktonic cells was quantified by recording the OD_600_, and then the planktonic cells and spent media were discarded and the remaining biomass was rinsed three times with distilled water. The plates were stained with CV and were quantified, as described above, while the metabolized TTC dye (appeared red in the wells) was resuspended in methanol and the A_500_ was recorded on a plate reader. The amount of biofilm inhibition was calculated relative to the amount of biofilm grown in the absence of peptide (defined as 100%) and the media sterility control (defined as 0%). Results from at least three separate biological replicates were averaged.

### 2.7. Effect of Media on Triphenyl Tetrazolium Chloride Metabolism

The effect of media composition on TTC metabolism was assessed using the same experimental setup, as described in the inhibition assay, with TTC (final concentration 0.05%) added to each well at the start of the experiment. The plates were incubated overnight at 37 °C under static conditions to allow for bacterial growth and biofilm maturation. The following day, the unadhered cells and the spent medium were discarded and the adhered biomass was rinsed three times with distilled water. The metabolized TTC dye (appeared red in the wells) was resuspended in 100 μL methanol with gentle mixing, and the absorbance at 500 nm was recorded on a microplate reader. Results represent the average of three separate biological replicates for each bacterial strain.

### 2.8. Quantification of Adhered Biofilm Cells in Microtitre Plates

To measure the amount of live cells that were adhered within the well of a microtitre plate, biofilms were grown overnight using the same procedure that was described for the inhibition assay. The following day, the wells were rinsed three times with distilled water, and the adhered biofilms were dislodged by sonication using a Thermo Fisher Scientific brand Model 120 Sonic Dismembrator, equipped with a 5/64” Microtip . Specifically, 200 µL LB was added to the wells containing *Pseudomonas* strains PAO1 and PA14 and sonicated for 20 s at a probe intensity of 35%. For the MRSA samples, 100 µL LB was added to the wells, followed by 20 s of sonication at a 20% probe intensity. These sonication parameters were used for each bacterial strain as they corresponded to the minimum time and probe intensity required to dislodge the adhered biomass without reducing the viability of the recovered CFUs. The resulting homogenized bacterial suspensions were serial diluted, plated onto LB-agar plates, and incubated overnight at 37 °C. The following day, the colonies were counted and the number of CFUs in each well was calculated.

## 3. Results

### 3.1. Biofilm Growth in Glass Tubes

**Figure 1:**
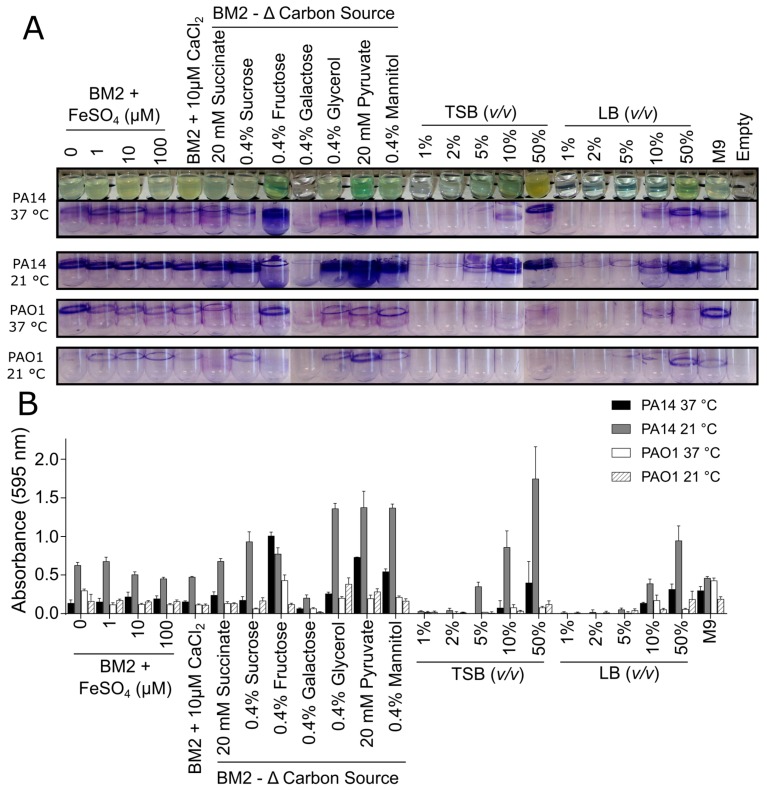
Effect of media composition and temperature on *Pseudomonas aeruginosa* biofilm growth when grown in glass tubes under static conditions. PA14 and PAO1 biofilms were grown under static conditions in glass culture tubes in modified basal medium 2 (BM2) minimal media or dilutions of rich media (tryptic soy broth (TSB) and Luria broth (LB)) at 37 °C (overnight) or 21 °C (two days). The following day, (**A**) the cultures were rinsed, stained with 0.1% crystal violet (CV), rinsed again, and imaged. The purple staining pattern represented biofilm biomass. (**B**) The CV was released by the addition of 70% ethanol and quantified by reading on a spectrometer. The samples were run in triplicate, averaged, and the error bars represent standard deviation.

The effect of medium composition on *P. aeruginosa* biofilm growth was first evaluated in glass tubes under static conditions. Biofilms formed by two lab strains of *P. aeruginosa*, PA14 and PAO1, were evaluated in several medium conditions, as well as at two different temperatures. Initial qualitative inspection of the CV staining pattern for both of these strains revealed a large variation in the level of biofilm adhered to the glass surface as well as a strong influence of temperature on the level of biomass that was stained by CV (Figure 1).

In general, strain PA14 exhibited more CV staining when compared to PAO1, indicating that PA14 might form more robust biofilms under these conditions (Figure 1A). Additionally, there appeared to be more CV-stained biomass for PA14 biofilms that were grown at 21 °C when compared to 37 °C (Figure 1A), indicating a temperature dependence for formation of strain PA14 biofilms, which was not evident for strain PAO1 biofilms. Resuspension of the bound CV dye in 70% ethanol and quantification by measuring the A_595_ further supported these observations since the absorbance values that were obtained for PA14 were consistently higher than those seen for PAO1 (Figure 1B). 

Biofilms are often grown in minimal media that might promote biofilm formation due to the limited availability of some nutrients. In particular, the use of a medium with a defined composition allows for researchers to probe the specific effects of nutrients on biofilm growth. Here we chose to use a common minimal medium, BM2, enabling the manipulation of the concentrations of additives, such as iron and calcium, as well as evaluating a variety of carbon sources. In the glass tube assay, supplementing BM2 medium with increasing amounts of Fe^2+^ had no impact on the amount of CV staining observed for PA14 and PAO1 biofilms (Figure 1). Likewise, the addition of 10 μM CaCl_2_ did not dramatically change the level of biomass that was stained under these conditions. Varying the carbon source, however, substantially affected the amounts of biofilm biomass deposited on the glass tube depending on the *Pseudomonas* strain and the type of carbon source used. For example, fructose, glycerol, pyruvate, and mannitol enhanced biofilm deposition and growth when compared to glucose, and this effect was particularly evident with strain PA14 (Figure 1). Conversely, *P. aeruginosa* samples in BM2 that were supplemented with 0.4% galactose resulted in very low levels of CV staining, as consistent with the lack of growth of *P. aeruginosa* in BM2 galactose (Figure 1A).

Two commercially available complex nutrient media, TSB and LB, were also tested at varying dilutions to examine whether the stresses from decreased nutrients in rich media promoted biofilm growth and accumulation. In the glass tube assay, a direct relationship was found between planktonic growth and CV staining. More dilute medium (1–2%) yielded little evidence of planktonic growth, and almost no CV-stained biomass, while increasing the medium strength resulted in enhanced planktonic growth and higher biofilm deposition with the level of planktonic growth in these samples (assessed by turbidity) largely mirroring the level of biofilm growth (Figure 1; see representative images of PA14 tubes grown at 37 °C). Overall, the results from these glass tube assays clearly demonstrated that the medium composition and the specific bacterial strain have a large impact on biofilm growth.

### 3.2. Biofilm Growth in Microtitre Plates

Due to our interest in developing a high throughput screening assay that is suitable for microtitre plates, we next tested the effect of media composition on biofilm growth and quantification in 96-well microtitre plates. Additional media conditions were evaluated, including varying the concentration of MgSO_4_ in the BM2 media, since decreasing Mg^2+^ concentrations have been shown to influence biofilm formation in *P. aeruginosa* [47]. In addition, an MRSAstrain was added [16,20] to permit the evaluation of medium-dependent biofilm formation by both Gram-negative and Gram-positive pathogens. As in the glass tube assays described above, bacterial cells from an overnight suspension that was grown in LB medium were inoculated into 100 μL of sterile media and the plates were incubated overnight at 37 °C under static conditions.

**Figure 2:**
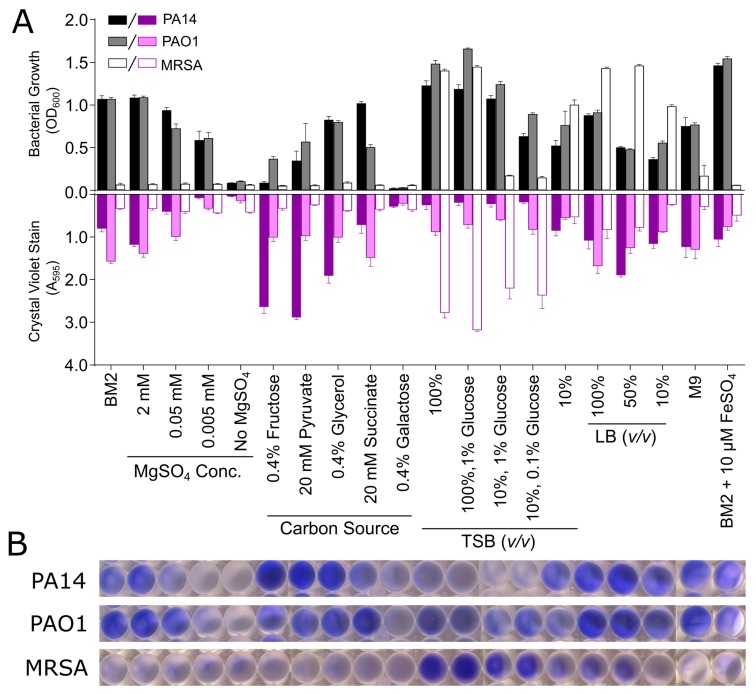
Effect of media composition on bacteria growth and CV staining for *P. aeruginosa* strains PAO1 and PA14 and *Staphylococcus aureus* MRSA biofilms grown in microtitre plates. PA14, PAO1, and MRSA biofilms were grown under static conditions at 37 °C overnight in 96-well microtitre dishes in modified BM2 minimal media or dilutions of rich media (TSB and LB). The following day, the optical density (OD_600_) of the plate was measured on a spectrometer (**A**, top graph, grey scale bars). The biofilm biomass was stained with 0.1% CV, dissolved in 70% ethanol and quantified at the absorbance of 595 nm (A_595_) (**A**, bottom graph, purple scale bars). (**B**) Representative wells were photographed to demonstrate typical results. The samples were run in triplicate and the recorded absorbance values were averaged with error bars representing standard deviation.

Prior to biomass quantification, the bacterial growth in each well was assessed by measuring the OD_600_ in each well. This revealed large differences in the level of growth that was supported by each medium and bacterial strain (Figure 2). In general, the *Pseudomonas* strains grew more readily in the BM2 minimal media conditions when compared to MRSA (Figure 2A). Interestingly, decreasing the Mg^2+^ concentrations in BM2 reduced the overall level of bacterial growth, while the effects of the various carbon sources were again highly variable, with only galactose resulting in little observable bacterial growth. Nearly all of the TSB or LB based conditions resulted in robust bacterial growth, with the only exception being MRSA grown in 10% diluted TSB, supplemented with glucose (Figure 2A).

Removing unbound bacteria from the microtitre plate and staining the adhered biomass with CV revealed a strong dependence of the level of biofilm growth on medium composition, as consistent with the results that were seen in the glass tube assay (Figure 2A). Images of representative wells containing the resuspended CV dye from each bacterial species are shown to illustrate the diversity of responses (Figure 2B). Again, a large variation between the *Pseudomonas* strains was observed, with PA14 tending to form more biofilm biomass, as quantified by stronger CV staining in general when compared to PAO1 (Figure 2A). Interestingly, several of the same trends could be seen under specific medium conditions. For instance, PA14 grown in BM2 fructose, pyruvate or glycerol, yielded strong CV staining compared to BM2 glucose. Conversely, in BM2 supplemented with succinate, PA14 supported higher planktonic growth than did strain PAO1, but had less CV staining. As before, BM2 containing galactose did not allow for either bacterial or biofilm growth, as evidenced by low absorbance values for both of the measurements (Figure 2A). Contrary to the concept that minimal medium might promote biofilm formation, quite robust biofilm growth was observed in all dilutions of LB, while TSB was somewhat less capable of supporting *Pseudomonas* biofilm formation, especially for strain PA14 (Figure 2A).

The level of CV staining that was obtained for MRSA grown in the microtitre plates was generally much lower than that seen for the *Pseudomonas* strains, particularly for the samples prepared in modified BM2 media (Figure 2). This was to be expected, as many of the minimal medium conditions did not support strong MRSA growth in general. Samples that were prepared in TSB or LB largely supported bacterial growth for MRSA; however, differences in adhered biomass were observed under these conditions as robust CV staining occurred in samples that were prepared in TSB, while LB media at various dilutions resulted in weaker CV staining. Full strength TSB supplemented with 1% glucose resulted in similar biofilm growth when compared to TSB alone. Similarly, diluted TSB (10%) supplemented with either 1% or 0.1% glucose also resulted in high levels of CV staining, while 10% TSB alone did not (Figure 2A). Interestingly, these diluted 10% TSB samples supplemented with glucose demonstrated very low MRSA growth, as evidenced by reduced OD_600_ measurements, while the 10% TSB sample without any glucose supplementation had an OD_600_ value that was closer to that seen in full strength TSB. Glucose has previously been shown to play a role in biofilm formation by *S. aureus* and *Staphylococcus epidermidis* [43], and the results from our screening assay support this finding, while also suggesting that this effect occurs under planktonic growth-limiting conditions. Overall the results in Figure 2 also indicated that there was no direct correlation between the level of bacterial growth and the amount of biofilm that was formed for any bacterium.

Tetrazolium salts are common reagents used in biological assays to assess the metabolic activity of living cells, since their energy driven reduction by cellular NADH leads to the appearance of coloured formazan salts. Thus, various tetrazolium-based dyes have also been used to quantify biofilms [36,37]. Here, we evaluated the use of triphenyl tetrazolium chloride (TTC) as a metabolic indicator of biofilm growth. In this case, the level of biofilm viability was estimated colorimetrically by quantifying the production of reduced 1,3,5-triphenylformazan that was generated by metabolically active biofilm cells, which appeared to be red in the sample.

**Figure 3:**
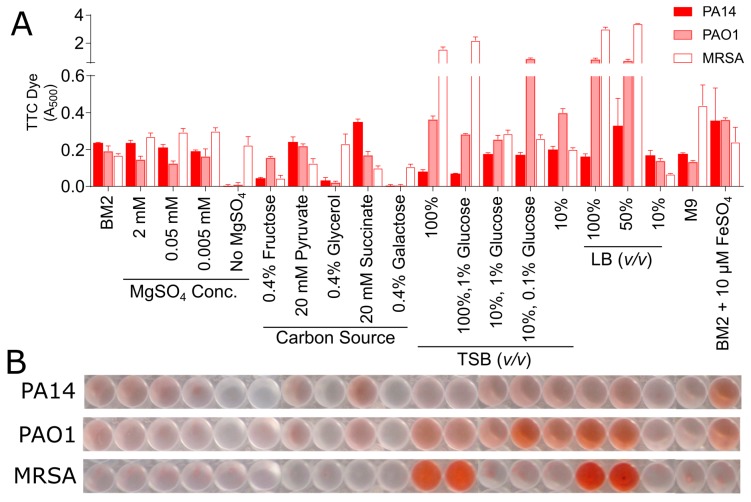
Effect of medium composition on TTC metabolism of *P. aeruginosa* strains PAO1 and PA14 and *S. aureus* MRSA. PA14, PAO1, and MRSA bacteria were mixed with the TTC dye and grown under static conditions overnight at 37 °C in 96-well microtitre dishes in modified BM2 minimal medium as well as dilutions of rich media (TSB and LB). (**A**) The following day, the plate was rinsed and the TTC dye was released from adhered cells by the addition of 100% methanol and quantified at A_500_. (**B**) Representative wells were photographed to demonstrate typical results. The samples were run in triplicate, averaged, and the error bars represent standard deviation.

To evaluate the effect of media composition on TTC metabolism in biofilms, samples were prepared similarly to those that were described for the CV staining methods described above. In this case, TTC was added to the bacterial suspension when the medium was inoculated and the appearance of the red 1,3,5-triphenylformazan end product in the adhered biomass was quantified spectrophotometrically after overnight incubation. A large effect of the media composition was again observed for many of the sample conditions, which is analogous to the CV assay (Figure 3B), although the specific results from the two different detection methods differed. Several of the BM2 conditions yielded low but reproducible levels of reduced TTC with A_500_ values ranging from ~0.1 to 0.25 absorbance units (Figure 3). Moreover, when grown in commercially prepared media, such as TSB and LB, several sample conditions caused significant increases in the TTC dye conversion, but again this was medium and strain specific. These results further underscore the need to choose and understand the influence of the selected growth medium to enable the quantification of biofilm growth in microtitre plates.

We next sought to establish whether there was any correlation between the level of bacterial growth in a microtitre plate, CV staining, the amount of TTC dye metabolized, and the number of CFUs that were found in the adhered biomass on the surface of the microtitre well. Based on the results from our in vitro screening assays, we evaluated the growth of PA14, PAO1, and MRSA biofilms in four different growth media, BM2, BM2 with 0.4% fructose as a carbon source, TSB supplemented with 1% glucose, and 10% TSB supplemented with 0.1% glucose. These conditions were selected, as they would provide a suitable cross section of media conditions, which gave varied responses depending on the bacterial strain and the assay being evaluated.

**Figure 4:**
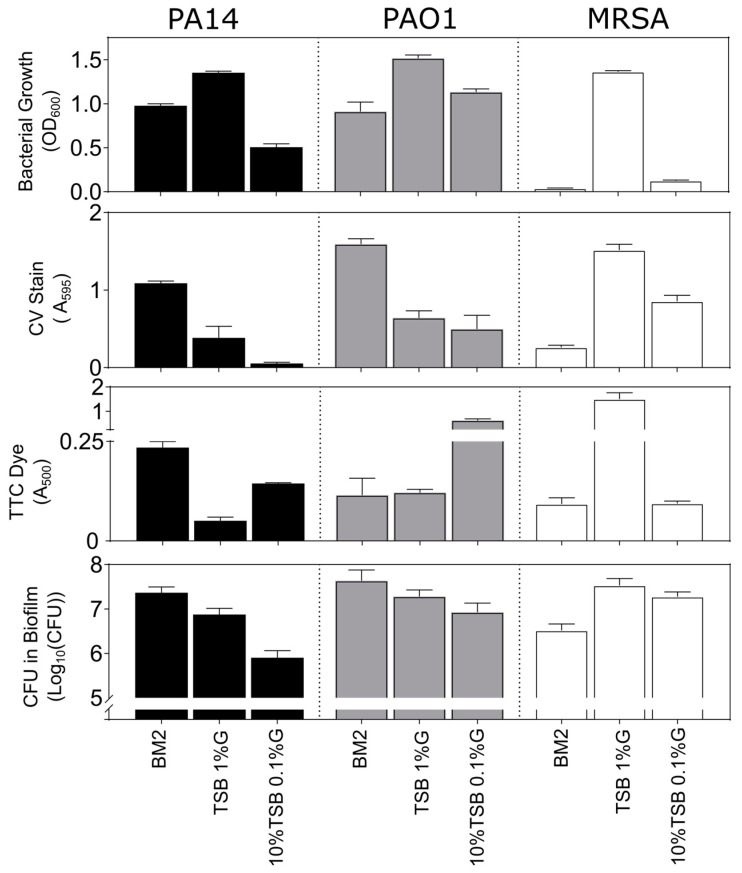
Relationship between bacterial growth, CV stain, TTC metabolism and CFUs in biofilms grown in microtitre plates. *P. aeruginosa* strains PA14 and PAO1, and *S. aureus* MRSA biofilms were grown under static conditions overnight at 37 °C in 96-well microtitre dishes in BM2 glucose minimal medium, TSB with 1% glucose (G) and 10% TSB with 0.1% glucose. Top Row: the following day, the OD_600_ of each well of the plate was assessed to determine growth. Second row: the adherent cells after rinsing were stained with 0.1% CV that was dissolved in 70% ethanol and quantified at A_595_. Third row: wells containing the TTC dye were rinsed and the TTC dye associated with adherent cells was released by the addition of 100% methanol and quantified at A_500_. Bottom row: the adherent cells after rinsing were homogenized and plated for overnight growth and the resulting CFU’s were counted the following day. All samples were run in triplicate, averaged, and the error bars represent the standard deviation.

As for the above assays, both *Pseudomonas* strains PA14 and PAO1 exhibited reasonably good bacterial growth in the order TSB/1% glucose > BM2 glucose > 10%TSB/0.1% glucose for PA14 and TSB/1% glucose > 10%TSB/0.1% glucose ≈ BM2 glucose for PAO1 (Figure 4). Conversely, the Gram-positive MRSA grew well only in TSB supplemented with 1% glucose. Generally speaking, the CV staining of biomass and the assessment of Log_10_CFU in the adhered biofilms demonstrated the same trends for *P. aeruginosa* strains with BM2 glucose > TSB/1% glucose > 10%TSB/0.1% glucose. Interestingly, the results for growth in 10%TSB/0.1% glucose for PA14 revealed that, despite a lack of CV staining, appreciable numbers of bacteria (~8 × 10^5^) were still adhered to the surface of these wells. This could indicate that CV staining might require a minimum threshold of adhered biofilm cells to allow for sufficient dye binding, further emphasizing the need to optimize growth conditions for sufficient biofilm growth.

In the TTC assay, metabolic activity provided a somewhat different perspective. The results for the two media supporting the largest colony counts for PA14 revealed the same order of metabolism BM2 glucose > TSB/1% glucose with modest TTC metabolism readings of between 0.15–0.25 absorbance units, while PAO1 grown under these conditions yielded similar TTC absorbance readings (Figure 4). Results for the 10%TSB/0.1% glucose conditions were anomalous as strain PAO1 biofilms, and to a lesser extent strain PA14, grown in this media resulted in strong A_500_ readings, despite evidence from CV staining and colony counts that would suggest modest biofilm formation under these conditions (Figure 4).

For MRSA biofilms, growth in TSB containing glucose resulted in higher CV and bacterial counts with the TSB/1% glucose condition supporting higher growth when compared 10%TSB/0.1% glucose, while also yielding ~60% higher adhered biomass according to CV staining (Figure 4). This also correlated somewhat with results using the metabolic TTC stain, although the disparity in metabolism was in this case very substantial with TSB/1%glucose > 10%TSB/0.1% glucose with the former giving far higher absorbance values than any other evaluated condition.

Results were also obtained for growth in BM2 fructose (Figure S2). As reported above, planktonic growth of *P. aeruginosa* was almost nonexistent under these conditions, as revealed by low OD_600_ values. However, robust biofilm growth was revealed by very strong CV staining and high CFU counts suggesting that bacteria can grow better as biofilms in this medium. Conversely, there was almost no metabolic activity, as revealed by TTC staining in biofilms that were grown in BM2 fructose, perhaps indicating limitations in biofilm metabolism in this medium. Overall, these data point to CV staining being a more reliable method of assessing biofilm development when compared to metabolic stains in adherent biofilm assays.

### 3.3. Effect of Media on *Pseudomonas aeruginosa* Biofilms Grown under Flow Conditions

**Figure 5:**
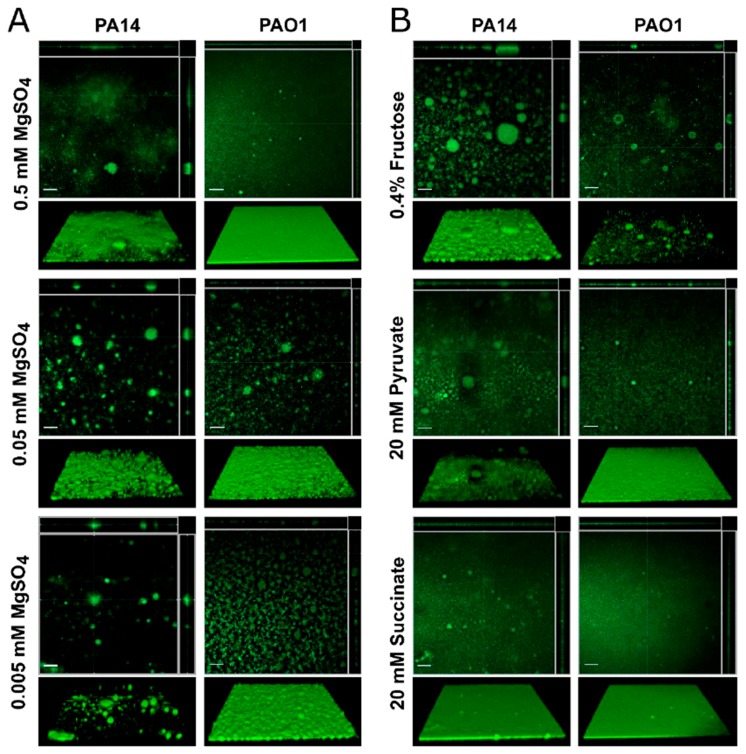
Effect of medium composition on *P. aeruginosa* strains PA14 and PAO1 biofilms grown in flow cells. (**A**) Biofilms were grown at 37 °C for three days under flow conditions in BM2 glucose minimal medium modified with varying MgSO_4_ concentrations. After three days, the chambers were flushed with BM2 medium to remove planktonic cells and the adhered biomass was stained with the DNA stain SYTO-9. (**B**) Biofilms were also grown in BM2 medium with 0.5 mM MgSO_4_ and varying carbon sources. Biofilms were imaged by confocal microscopy on a Zeiss LSM 800 microscope at 20× magnification. Three-dimensional images of the adhered biofilms were captured as a stack of images along the *z*-axis and compiled into top down orthogonal reconstructions (upper panels) using the Zen Software package and the 3D reconstructions (lower panels) using the ImageJ software package Fiji [45,46]. The white scale bar in the orthogonal displays represents 50 μm. Images shown are representative of the biofilms found throughout the flow cell chamber and conditions were evaluated at least in duplicate.

To confirm these data, we evaluated the effect of media composition on *Pseudomonas* biofilms grown in flow cells over a period of days. Specifically, the effect of altering the MgSO_4_ concentration or varying the carbon source in BM2 was evaluated for *P. aeruginosa* strain PA14 and PAO1 biofilms that were grown in flow cell chambers. In the microtitre experiments described above, decreasing amounts of MgSO_4_ in the BM2 media resulted in reduced bacterial growth (OD_600_), as well as decreased CV staining (Figure 2). Previous publications have also demonstrated that Mg^2+^ ions influence the architecture of *Pseudomonas* biofilms when grown under flow conditions [47,48]. This was confirmed here, since MgSO_4_ had a substantial effect on the architecture of PA14 biofilms that are grown under flow conditions (Figure 5). Using our standard BM2 glucose containing 0.5 mM MgSO_4_, PA14 formed a biofilm mat on the glass coverslip of the flow cell with few large mushroom structures. However, as the concentration of MgSO_4_ was reduced to 0.05 and 0.005 mM, the biofilm architecture became populated with larger and more numerous mushroom-shaped biofilm structures appearing (Figure 5A).

Carbon sources also dramatically influenced biofilm growth in microtitre assays that are based on both CV staining and TTC metabolism. Similarly varying the carbon source in BM2 minimal medium also had a large effect on *Pseudomonas* biofilm architecture when being grown under conditions of continuous flow. Specifically, PA14 grown in BM2 0.4% fructose as the carbon source yielded robust biofilms with numerous large mushroom shaped biofilms (Figure 5B). Likewise, BM2 pyruvate supported biofilm growth, similar to the BM2 glucose. In BM2 succinate, strain PA14 formed a uniform bacterial mat with a few small punctate structures being distributed across the biofilm surface (Figure 5B).

In general, PAO1 biofilms, when compared to PA14, showed greater consistency in CV staining and TTC metabolism when grown in various media in the microtitre assays. This trait carried through into the flow cell experiments since strain PAO1 biofilms formed robust bacterial mats on the flow cell surface but the overall architecture of the resulting biofilm was not as pronounced and many of the three-dimensional features were dampened when compared to those that were formed by PA14. For example, the surface of the PAO1 biofilms became rougher with several small mushroom shapes being dispersed across the surface of the biofilm mat as the concentration of MgSO_4_ in the medium was reduced, but these structures were smaller in comparison to the PA14 structures (Figure 5A). Similarly, PAO1 formed more structures in BM2 fructose, but most of these were much smaller than those that were seen in PA14 under the same conditions (Figure 5B). When grown in BM2 pyruvate or BM2 succinate, a robust mat of adhered biomass was observed, which was similar to the architecture of PA14 that was grown in BM2 succinate (Figure 5B).

### 3.4. Biofilm Inhibition and Eradication Assays in Microtitre Plates

The driving force behind this work was to establish screening assays that could be used to assess the antibiofilm activity of various compounds, particularly antibiofilm peptides. Since, clinically speaking, there is an urgent need to identify compounds that can treat existing biofilm infections, we sought to evaluate the capacity of a compound to eradicate, disperse, or inhibit the growth of a preformed biofilm that was already established within the wells of a microtitre plate. To illustrate the type of data that can be obtained from this type of assay and to compare to the more conventional assays in which biofilm development is assessed, the antibiofilm activity of various antibiotics as well as three synthetic antibiofilm peptides was evaluated under both inhibition (addition prior to biofilm initiation) and eradication (addition after biofilm formation) conditions against *P. aeruginosa* PAO1 and MRSA biofilms. For the eradication assay, since we utilized growth conditions, in which both methods gave analogous results (Figure 4), we elected to assess both adhered biomass using CV stain as well as measuring the metabolic activity of the biofilm sample using TTC dye. This enabled the better interpretation of the eradication assay results.

**Figure 6:**
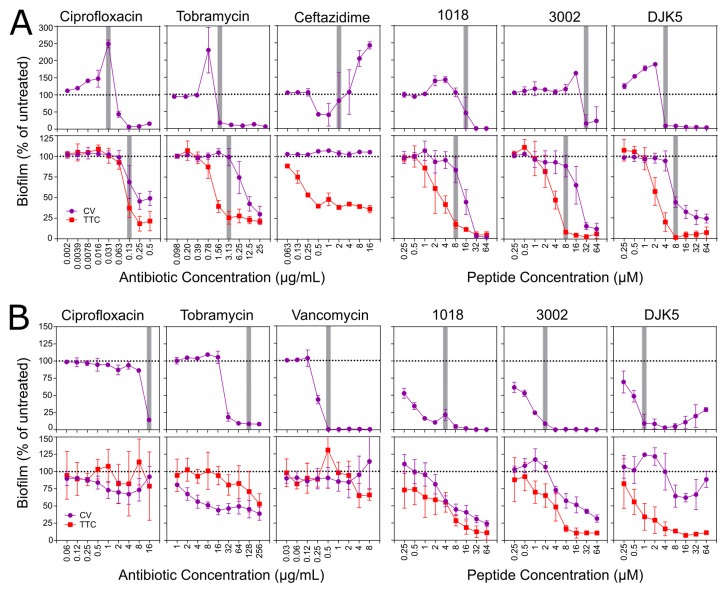
Effect of antibiotics and antibiofilm peptides on biofilm growth evaluated under both inhibition and eradication conditions. (**A**) *P. aeruginosa* PAO1 biofilms were grown in BM2 glucose minimal medium (**B**) *S. aureus* MRSA biofilms were grown in 10% TSB supplemented with 0.1% glucose. Inhibition is shown in the upper panels and eradication assays in the lower panels. The adhered biomass within the wells was quantified by CV staining (purple lines) while the metabolic activity in the preformed biofilm samples was quantified by conversion of TTC dye to the red formazan end product (red lines). The vertical gray bars indicate the concentration of antibiotic or peptide that resulted in greater than 90% reduction in bacterial planktonic growth as measured by recording the OD_600_ in each well prior to rinsing the spent media. Data represents the average ± standard deviation (SD) of at least three biological replicates.

When being evaluated under inhibitory conditions, biofilms that were formed by both MRSA and PAO1 showed similar trends in the presence of antibiotics and peptides. The inhibitory effects of the antibiotics towards PAO1 biofilms indicate the inhibition of biofilm growth that occurred at concentrations higher than the concentration that inhibited bacterial growth. Interestingly, treatment with ciprofloxacin and tobramycin enhanced CV staining at concentrations that were immediately below the inhibitory concentration (Figure 6A, upper panel), as is consistent with previous observations of tobramycin-treated *P. aeruginosa* biofilms at sub-inhibitory concentrations [49]. Ceftazidime was exceptional as a tendency to inhibit adhered biomass by ~50% at 0.5 and 1 µg/mL was superseded by a concentration-dependent increase in biofilm formation at higher concentrations, with 2.5-fold enhanced CV staining observed at 16 µg/mL compared to untreated controls (Figure 6A). Conversely, each of the peptides strongly inhibited biofilm formation at concentrations at or above the concentration that inhibited bacterial growth in these assay conditions.

With regard to the eradication assays that assessed treatment of one-day old pre-formed biofilms, none of the antibiotics showed the complete eradication of CV-stained *P. aeruginosa* biomass, nor did they completely inhibit the metabolic activity of the adhered biofilms (Figure 6A lower panel), even at concentrations 4–8-fold greater than the concentration that inhibited bacterial growth overall. Ciprofloxacin and tobramycin treated samples led to an ~50% reduction in CV staining and a ~75% reduction in TTC metabolism when compared to control samples, respectively. Ceftazidime treatment of PAO1 biofilms did not reduce the level of CV staining at any concentration evaluated, and only reduced the level of metabolic activity by ~60% at concentrations higher than 0.5 μg/mL. In contrast, all three peptides reduced both the adhered biomass and the metabolic activity of the PAO1 biofilms in a dose-dependent manner. Based on CV staining, 3002 and DJK-5 treated samples reduced the amount of CV that was stained by up to 85% when compared to untreated controls, while biofilms that were treated with greater than 32 μM 1018 reduced the adhered biomass to near baseline levels (Figure 6A). The metabolic activity of the peptide treated samples revealed that all three peptides largely inhibited the metabolic activity of PAO1 biofilms at concentrations that were greater than 8 μM, particularly 3002 and DJK-5 treated samples that approached baseline levels. All of the metabolic activities were reduced at concentrations lower than those that were required to inhibit biomass, suggesting the possibility that residual CV staining might be at least in part due to dye binding to dead cells.

For MRSA inhibition assays, the amount of CV staining was consistently low at antibiotic or peptide concentrations that were higher than the concentration that inhibited bacterial growth (Figure 6B, upper panel). This is as we originally anticipated, since concentrations that inhibit planktonic growth would, by extension, prevent a critical accumulation of bacterial cells that could adhere and form a mature biofilm. The minor exceptions to this were tobramycin-treated samples that never quite reached baseline levels of CV staining, as well as MRSA treated with DJK-5 that showed a slight increase in CV staining at peptide concentrations that were higher than 16 μM (Figure 6B).

Treatment with ciprofloxacin, tobramycin, or vancomycin of pre-formed MRSA biofilms grown in 10%TSB/0.1%glucose did not cause major decreases in either the level of CV staining or the amount of TTC metabolism that was observed (Figure 6B, bottom panel). Only tobramycin treated samples saw a ~50% reduction in both parameters, and this was only observed at antibiotic concentrations approaching 256 μg/mL. MRSA biofilms that were treated with the other antibiotics were similar to untreated control samples at all of the antibiotic concentrations evaluated. This is consistent with the notion that bacterial cells within a biofilm are adaptively resistant to antibiotics. Treatment of the MRSA biofilms with antibiofilm peptides revealed a different trend, since the peptides caused dose dependent decreases in both the amount of adhered biomass as well as the metabolic activity of the cells within the biofilm (Figure 6B). For both 1018 and 3002, the level of CV stain was reduced by up to 75% when compared to untreated samples, while the amount of TTC dye metabolized approached a 90% reduction when compared to control samples. This indicates that these synthetic peptides inhibited the maturation of preformed MRSA biofilms and suggests that many of the bacterial cells within the adhered biomass have significantly reduced metabolic activity. Interestingly, high concentrations of DJK-5 resulted in an increase in the level of CV staining, which is a phenomenon that was also observed in the inhibition assay. This was likely not due to enhanced biofilm growth under these conditions, but instead it is probably the result of an unknown effect of DJK-5 on MRSA cells at high concentrations. Critically, none of the peptide-treated MRSA biofilms demonstrated reduced planktonic bacterial growth in the wells where the biofilms were inhibited.

The use of luminescent or fluorescent reporters to quantify biofilm growth has also been explored to evaluate antibiofilm compounds in a high-throughput fashion. For example, a luminescence-based screening approach successfully identified small molecule inhibitors of *P. aeruginosa* biofilms [50]. A high-throughput microfluidic approach was also described using green-fluorescent protein (GFP)-expressing *Pseudomonas,* coupled with propidium iodide staining to quantify biofilm growth and cell viability [51]. Therefore, we also explored the use of genetically-modified bacteria that constitutively expressed either GFP proteins or the *luxCDABE* luciferase reporter cassette to detect adherent biofilms using fluorescence or luminescence. Using the same basic set-up for biofilm inhibition and eradication assays described above, we found that these two detection methods were highly reproducible and they resulted in comparable antibiofilm activities for the antibiofilm peptides 1018 and DJK-5 when compared to the CV and TTC dye based assays (Figure S3). The caveat to using these types of assays is that they require the use of genetically modified bacteria and specialized plate readers. This prevents the assessment of the biofilm forming capacities of clinical isolates or bacterial species for which genetic tools are lacking. In addition, these assays did not simplify the detection procedure, and in fact required longer growth times to obtain reliable fluorescence and luminescence readouts from the adhered biofilm cells. Therefore, in our opinion, there was no distinct advantage offered by fluorescent or luminescent bacterial strains that would justify their use over CV or TTC dyes.

## 4. Discussion

There have been numerous calls for a standardized method to study biofilm formation in vitro [52,53]. Several methods have been proposed in the literature to measure biofilm growth and to evaluate the antibiofilm activity of compounds in a high throughput fashion, including the Calgary biofilm device [32], the BioFilm Ring Test [54], and the xCELLigence real time cell analyzer [55]. Each of these methods have successfully demonstrated the capacity to screen for antibiofilm compounds; however, they require specific equipment that may not be available to every researcher, and they generally are used to test biofilm inhibition rather than eradication. In addition, the Calgary biofilm device requires the use of multiple sterile microtitre dishes to accommodate the various rinsing and treatment stages, and relies on viable cell counting for experimental validation [56], which adds additional costs and can be cumbersome when testing large compound libraries. In the present work, we have outlined high throughput procedures to analyze the effects of antibiofilm peptides on both inhibition and eradication of biofilms that considers reproducibility, ease of use, and cost. Our findings also emphasize that future adopters of these methods must understand the nature of their organism of interest and appreciate the influence that their growth conditions and the experimental setup will have on the CV and TTC staining procedures. 

The effects of media on biofilm growth have been previously explored for various types of bacteria [43,57,58,59,60,61]. Many of these studies evaluated a limited number of medium conditions and most sought to evaluate the strain and mutant dependence on biofilm formation rather than media composition specifically. The results from the CV screening assays that are presented here clearly support a strong influence of media composition on the amount of biofilm that can be quantified. Differences in staining in specific media might incompletely mirror actual biofilm levels, since CV will stain even dead cells or it could also reflect different biofilm mechanisms that are activated or are repressed under specific conditions. For instance, it was striking to see that high levels of CV staining persisted in MRSA samples that were grown in 10% TSB conditions, supplemented with glucose when compared to full strength TSB media, even though there was a large discrepancy that was seen in the overall bacterial growth between these conditions (Figure 2). Exploring the mechanisms underlying these differences in biofilm staining is beyond the scope of the present work, but it underscores the importance of understanding the molecular underpinnings of these differences which could give important insights into biofilm formation for a given organism. Moreover, an experimental approach, such as the one described here, could be extended to screening mutant libraries of a given bacterial strain under various media conditions to look for genes that consistently contribute to biofilm formation or identify genes that promote biofilm formation under specific conditions. Such a strategy would yield further mechanistic insights into how biofilms form for individual bacterial species and could identify unexplored drug targets that could be exploited for creating future antibiofilm therapeutics.

It is important to emphasize that any level of CV staining (or other biofilm quantification method) must reflect adhered bacterial cells within the well of the microtitre plate, and therefore this situation should be considered as a biofilm. The architecture and composition of these biofilms may change, depending on the growth conditions of the sample, but any effect of antimicrobial compounds that reduces the number or viability of these adhered cells can be interpreted as antibiofilm activity. Conversely, an apparent lack of activity in either of these assays does not necessarily mean that a compound of interest is devoid of antibiofilm activity. For instance, high levels of CV staining might reflect the creation of cell debris, which remain bound to the surface of a well, which would look like adhered biofilms, even if they were not viable. Similarly, a reduction in metabolic activity could reflect cell lysis, the reduction in energy generation due to close packing of cells (as seen at the base of biofilm colonies) or biofilm dispersal. For this reason, it is important to adopt a range of methods during primary and secondary screening to identify potential antibiofilm compounds.

Several studies have sought to compare various biofilm detection methods in an attempt to find an optimal assay [35,52,62,63,64,65]. In one of the more comprehensive studies, six methods were compared to analyze different characteristics of bacterial life in biofilms in five different test organisms [63]. CV staining was used to identify biomass, SYTO 9 and dimethylene blue labelling for nucleic acid within the polymeric matrix and inside of living and dead bacteria to measure biomass, the metabolism of fluorescein diacetate, the soluble tetrazolium dye 2,3-bis(2-methoxy-4-nitro-5-sulfophenyl)-5-[(phenylamino)carbonyl]-2*H*-tetrazolium hydroxide (XTT), and resazurin were used as a measure biofilm viability, and dimethyl methylene blue was used to identify sulphated polysaccharides within the polymeric matrix as an alternative assessment of biomass. This study revealed large differences between the assays, and the authors decided that some of the assays were less suitable than others, although only one type of medium was evaluated for each bacterial species. For instance, the authors approved of using soluble tetrazolium dyes to measure biofilm formation, but recommended against using XTT due to cost. Fortunately, the TTC dye that was used in our experimental setup is a considerably cheaper alternative, and it therefore fulfills our requirement to keep reagent costs to a minimum. Interestingly, the authors concluded that the CV assay was inappropriate for measuring *Pseudomonas* biofilms due to the large variations in the stained biomass between replicates, and instead recommended the metabolic dyes as the most reliable quantification methods for biofilm growth. In contrast, our analysis of PA14 and PAO1 in CV staining assays showed little variation among the experimental replicates (Figure 2), and medium-dependent disparities in metabolic staining (Figure 4; Figure S2). Thus it is possible that the assay conditions in that study [63], particularly the media composition, played a role in the variability of CV staining.

Through our study and in examining other published results, it would appear that standardizing assay conditions to quantify biofilm growth and assess antibiofilm activity, similar to MIC assays [22], might not be possible. While each of the dye detection methods that are described above can be used to provide valuable information regarding the specific aspects of biofilm growth, they are limited by the experimental setup and the specificity of biofilm formation for each bacterial strain being evaluated. Evidently, it appears that we must appreciate the complexity of biofilm growth when evaluating the results of antibiofilm screens. Thus, ultimately, it may be necessary to establish a series of biofilm type strains and recommended media to enable consistency of biofilm inhibition or eradication results. Therefore, we recommend that appropriate biofilm growth conditions should be established for each bacterial strain studied to ensure adequate detection and reproducibility using the dye-based detection method of choice. Furthermore, we suggest that comparing the absolute values of CV staining or absorbance readouts from other dyes (either between replicates or among research groups) is not appropriate, since these values are strongly influenced by the specific growth conditions, bacterial strain, and microplate reader used for quantification. Therefore, we also recommend normalizing the biofilm growth data in terms of a maximal response from untreated control wells (100%) and comparing this to a sterility control (0%). Such a strategy reduces the impact of the inherent variability using a non-specific dye, such as CV, and allows for comparisons between biofilm growth conditions for an individual strain or between separate bacteria species that are grown under distinct media conditions (such as *P. aeruginosa* PAO1 and *S. aureus* MRSA, as shown in Figure 6). The caveat to employing such a normalization procedure is that the dynamic range of the absorbance values that were obtained under specific sample conditions can vary substantially (see absorbance values for CV and TTC staining in Figure 4 and Figure S2). Therefore, one must appreciate that the growth conditions for which this dynamic range is small will contribute to the increased variability in the normalized data and the results should be interpreted appropriately.

Overall, high-throughput biofilm screening assays in microtitre plates, such as the biofilm inhibition and eradication assays described here, are essential tools that are desperately needed to identify candidate antibiofilm compounds to combat the problem of biofilm-associated infections. However, it is important to understand the limitations of each assay when interpreting the results and to take into account which aspects of the biofilm life cycle are being queried based on the experimental setup. Since biofilms are heterogeneous in their structure and organization, complementary approaches to assess antibiofilm activity should be used to confirm that the antibiofilm activity of identified “hits” from high-throughput screens are conserved in conditions that are clinically relevant. This paper thoroughly outlines two procedures that can be used to analyze biofilm inhibition and eradication by antibiofilm peptides, and are methodologies that we would like to see become the standard for the field.

## Supplementary Materials

The following are available online at http://www.mdpi.com/2218-273X/8/2/29/s1, Figure S1: Sample 96-well microplate layout for biofilm inhibition and eradication assays. Figure S2: Relationship between bacterial growth, CV stain, TTC metabolism and CFUs in biofilms grown in microtitre plates, Figure S3: GFP and Lux Reporters as detection tools for biofilm inhibition and eradication.
