# Supplementary material for: Critical Assessment of Methods to Quantify Biofilm Growth and Evaluate Antibiofilm Activity of Host Defence Peptides
Source: Biomolecules. 2018 May 21;8(2):29. doi: 10.3390/biom8020029 (PMC6022921; doi:10.3390/biom8020029)

Supplemental Materials for "Critical Assessment of Methods to Quantify Biofilm Growth and Evaluate Antibiofilm Activity of Host Defence Peptides"

Evan F. Haney†, Michael J. Trimble†, John T. Cheng, Quentin Vallé and Robert E.W. Hancock
† Joint-first authors; these authors contributed equally to this work.

Department of Microbiology and Immunology, Center for Microbial Diseases and Immunity Research, Department of Microbiology and Immunology, University of British Columbia, Vancouver, BC V6T 1Z4.

Supplemental Methods

Detection of Biofilms by Fluorescence or Luminesce

GFP expressing strains of PAO1 [1] and USA300 MRSA [2] as well as luminescent strains of PAO1 (strain H1001 from [3]) and USA300 [4] were used to assay biofilm detection by fluorescence or luminescence. Both biofilm inhibition and biofilm eradication were determined using methods described in the main text using the same medium conditions with a slight modification in the incubation time to allow for biofilm growth and maturation. Following inoculation with the bacteria (and with antimicrobial compound of interest in the inhibition assays), the plates were incubated for two days at 37 °C under static conditions. Incubation times shorter than this did not give reliable readouts of GFP fluorescence or luminescence. In the inhibition assays, after the first day of incubation, the spent medium was removed and 90 µl fresh medium containing 10 µl of peptide at 10X the final concentration or vehicle control was again added to each well. The following day, bacterial growth was quantified by recording the optical density at 600 nm in each well using a multi-modal microplate reader (Synergy H1 microplate reader, BioTek Instruments, Winooski VT). The planktonic cells and spent media were then discarded and the adhered biomass was rinsed three times with distilled water. The remaining adhered biomass was resuspended in 150 µl of 10% LB (*v/v*) in sterile PBS (pH 7.4) for the inhibition assays or 210 µl for the eradication assay by simple pipetting up-and-down and the luminescence or fluorescence signal was read immediately on a plate reader. The amount of adhered biofilm was quantified by measuring either the luminescence (Total white light, unfiltered) or fluorescence (eGFP, $\lambda ex = 488$ nm, $\lambda em = 530$ nm) of each sample, depending on the bacterial strain being evaluated. For both assays, the percent biofilm inhibition or eradication was calculated in relation to the amount of biofilm grown in the absence of peptide (defined as 100%) and the media sterility control (defined as 0% growth). Data from three separate biological replicates were averaged.

Supplemental References

1. Koch, B.; Jensen, L. E.; Nybroe, O. A panel of Tn7-based vectors for insertion of the gfp marker gene or for delivery of cloned DNA into Gram-negative bacteria at a neutral chromosomal site. *J. Microbiol. Methods* **2001**, 45, 187–195, doi:10.1016/S0167-7012(01)00246-9.
2. Kamenyeva, O.; Boularan, C.; Kabat, J.; Cheung, G. Y. C.; Cicala, C.; Yeh, A. J.; Chan, J. L.; Periasamy, S.; Otto, M.; Kehrl, J. H. Neutrophil recruitment to lymph nodes limits local humoral response to *Staphylococcus aureus*. *PLoS Pathog*. **2015**, 11, e1004827, doi:10.1371/journal.ppat.1004827.
3. Lewenza, S.; Falsafi, R. K.; Winsor, G.; Gooderham, W. J.; McPhee, J. B.; Brinkman, F. S.; Hancock, R. E. W. Construction of a mini-Tn5-luxCDABE mutant library in *Pseudomonas aeruginosa* PAO1: a tool for identifying differentially regulated genes. *Genome Res*. **2005**, 15, 583–589.
4. Plaut, R. D.; Mocca, C. P.; Prabhakara, R.; Merkel, T. J.; Stibitz, S. Stably luminescent *Staphylococcus aureus* clinical strains for use in bioluminescent imaging. *PLoS ONE* **2013**, 8, e59232, doi:10.1371/journal.pone.0059232.

Supplemental Figures

Supplemental Figure S1: Sample 96-well microplate layout for biofilm inhibition and eradication assays. Only the interior wells of the plate are used to mitigate the possible effect of evaporation of liquid from wells at the edge of the plate. The exterior wells are filled with sterile water or serve as a sterility control for the media of interest.

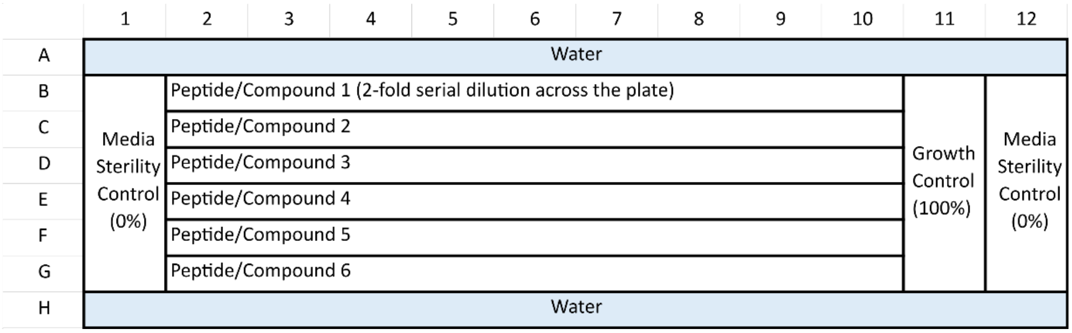

Supplemental Figure S2: Relationship between bacterial growth, CV stain, TTC metabolism and CFUs in biofilms grown in microtitre plates. *P. aeruginosa* strains PA14 and PAO1, and *S. aureus* MRSA biofilms were grown under static conditions overnight at 37 °C in 96-well microtiter dishes in BM2 glucose minimal medium or BM2 supplemented with 0.4% fructose. Top Row: the following day, the OD_{600} of each well of the plate was assessed to determine growth. Second row: the adherent cells after rinsing were stained with 0.1% CV that was dissolved in 70% ethanol and quantified at A_{595}. Third row: wells containing the TTC dye were rinsed and the TTC dye associated with adherent cells was released by the addition of 100% methanol and quantified at A_{500}. Bottom row: the adherent cells after rinsing were homogenized and plated for overnight growth and the resulting CFU's were counted the following day. All samples were run in triplicate, averaged, and the error bars represent the standard deviation.

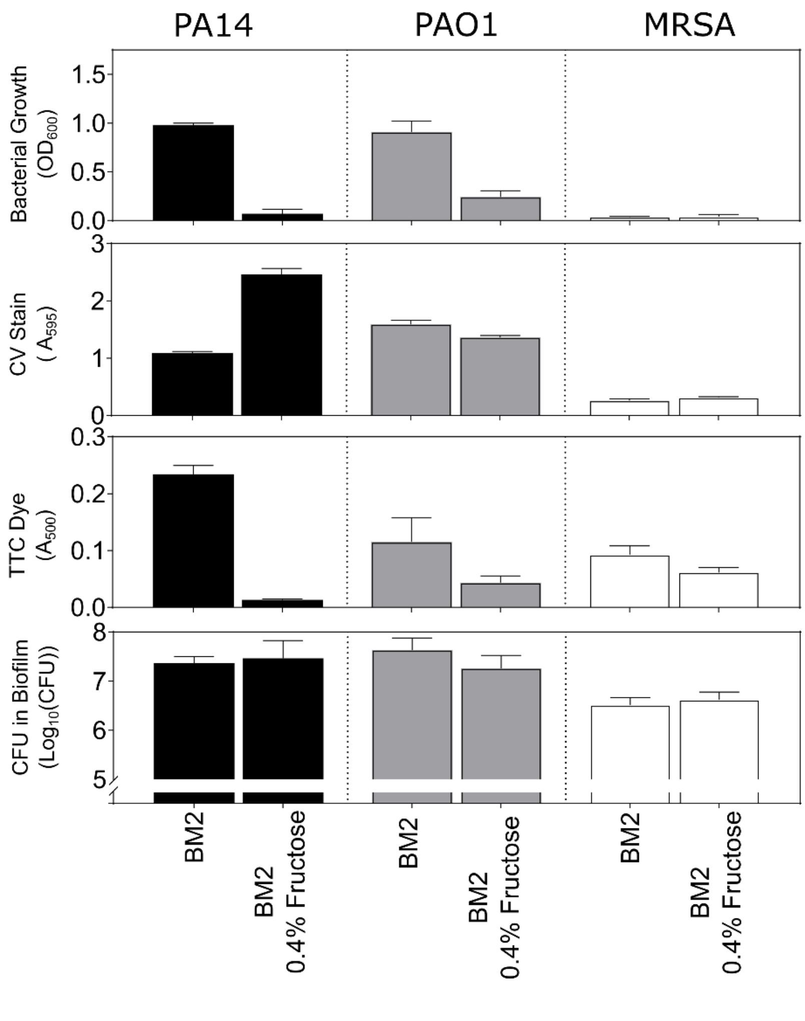

Supplemental Figure S3: GFP and Lux Reporters as detection tools for biofilm inhibition and eradication. The inhibition (Top panel) and eradication (Bottom panel) effect of two peptides, 1018 and DJK-5, were assessed on biofilms of GFP-expressing (green circles) or luminescent (blue squares) strains of (A) USA300 MRSA and (B) PAO1. Data are the average (± SD) of three biological replicates. The concentration of peptide that reduced bacterial growth in the well by 90% prior to rinsing is indicated by a green or blue vertical line, for GFP or lux bacteria respectively. Gray lines indicate that this concentration was identical for both strains.

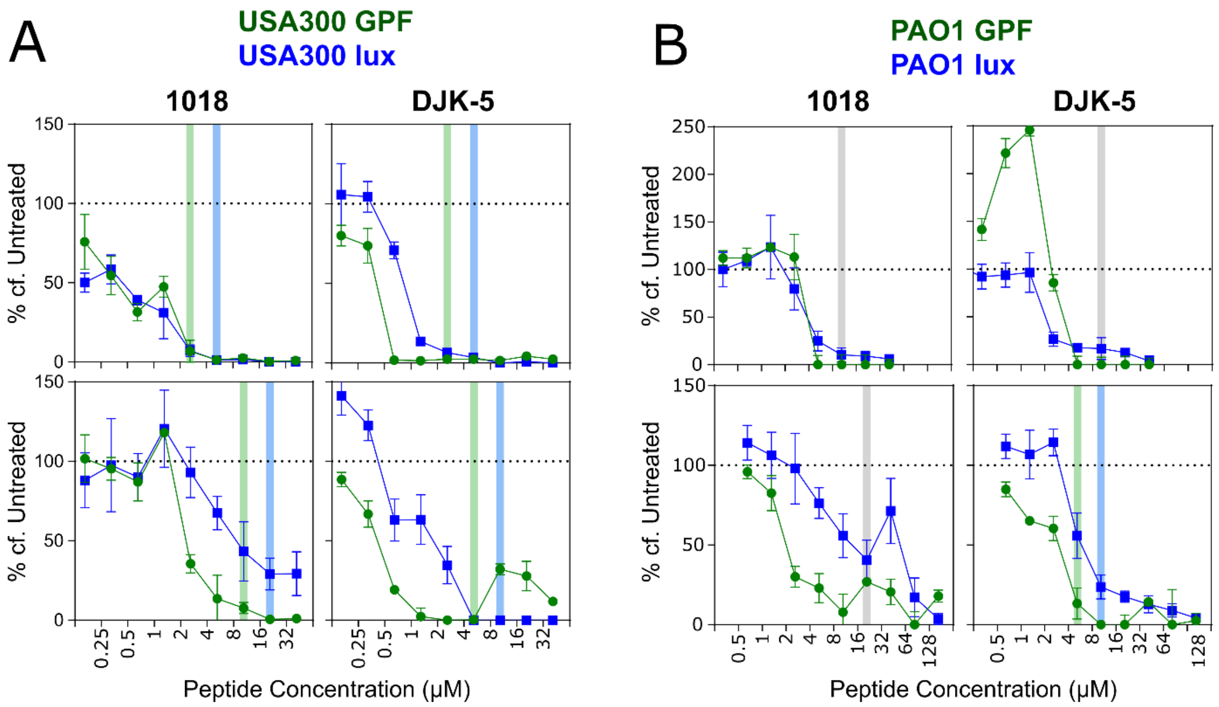

Supplement: Supplementary file 1 [file biomolecules-08-00029-s001.pdf]
